# 4-(4-Nitro­styr­yl)-*N*,*N*-diphenyl­aniline

**DOI:** 10.1107/S1600536812023719

**Published:** 2012-05-31

**Authors:** Zhi-Wen Zhang, Yan-Qiu Liu, Yu-Chao Zhu, Jie-Ying Wu

**Affiliations:** a Deparment of Chemistry, Anhui University, Hefei 230039, Peoples Republic of China, Key Laboratory of Functional Inorganic Materials, Chemistry, Hefei 230039, People’s Republic of China

## Abstract

In the triaryl­amine group of the title compound, C_26_H_20_N_2_O_2_, the N atom adopts an approximately trigonal–planar geometry, lying 0.046 (5) Å from the plane *P* defined by its three neighbouring C atoms; the benzene and two terminal phenyl rings are twisted by 37.4 (1), 31.4 (1) and 47.8 (1)°, respectively from plane *P*. In the *trans*-stilbene fragment, the two benzene rings form a dihedral angle of 31.3 (1)°. In the crystal, weak inter­molecular C—H⋯O inter­actions link the mol­ecules into ribbons in [100].

## Related literature

For a related structure, see: Yang *et al.* (2003 ▶). For background to push–pull chromophores, see: Marder *et al.* (1991 ▶); Reinhardt *et al.* (1998 ▶).
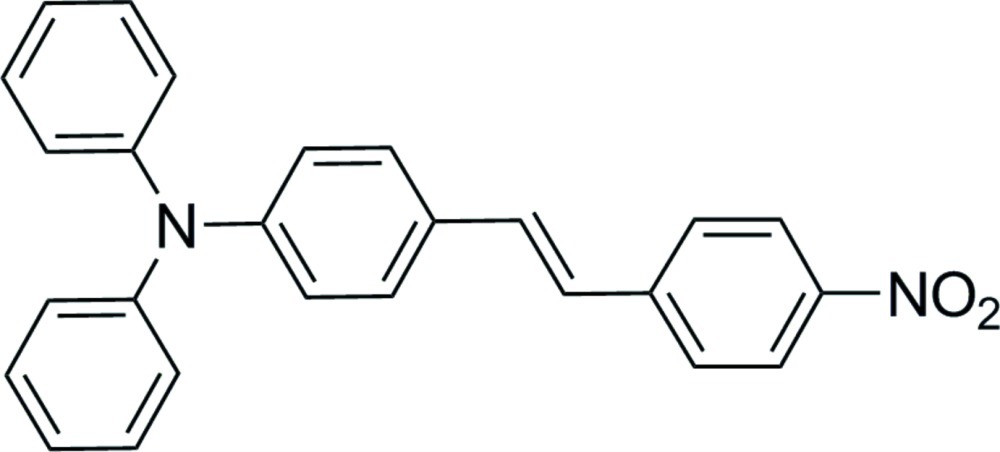


## Experimental

### 

#### Crystal data

C_26_H_20_N_2_O_2_
*M* *_r_* = 392.44
Monoclinic, 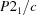

*a* = 8.4884 (3) Å
*b* = 8.9834 (3) Å
*c* = 27.0880 (8) Å
β = 96.500 (2)°
*V* = 2052.31 (12) Å^3^
*Z* = 4
Mo *K*α radiation
μ = 0.08 mm^−1^
*T* = 298 K
0.30 × 0.20 × 0.20 mm

#### Data collection

Bruker SMART CCD area-detector diffractometer
Absorption correction: multi-scan (*SADABS*; Sheldrick, 1996 ▶) *T*_min_ = 0.976, *T*_max_ = 0.984
7675 measured reflections
3606 independent reflections
2213 reflections with *I* > 2σ(*I*)
*R*_int_ = 0.026

#### Refinement

*R*[*F* ^2^ > 2σ(*F* ^2^)] = 0.057
*wR*(*F* ^2^) = 0.185
*S* = 1.04
3606 reflections
272 parameters
7 restraints
H-atom parameters constrained
Δρ_max_ = 0.44 e Å^−3^
Δρ_min_ = −0.20 e Å^−3^

### 

Data collection: *SMART* (Bruker, 2007 ▶); cell refinement: *SAINT* (Bruker, 2007 ▶); data reduction: *SAINT*; program(s) used to solve structure: *SHELXS97* (Sheldrick, 2008 ▶); program(s) used to refine structure: *SHELXL97* (Sheldrick, 2008 ▶); molecular graphics: *SHELXTL* (Sheldrick, 2008 ▶); software used to prepare material for publication: *SHELXTL*.

## Supplementary Material

Crystal structure: contains datablock(s) I, global. DOI: 10.1107/S1600536812023719/cv5302sup1.cif

Structure factors: contains datablock(s) I. DOI: 10.1107/S1600536812023719/cv5302Isup2.hkl

Supplementary material file. DOI: 10.1107/S1600536812023719/cv5302Isup3.cml

Additional supplementary materials:  crystallographic information; 3D view; checkCIF report

## Figures and Tables

**Table 1:** Hydrogen-bond geometry (Å, °)

*D*—H⋯*A*	*D*—H	H⋯*A*	*D*⋯*A*	*D*—H⋯*A*
C15—H15⋯O1^i^	0.93	2.58	3.481 (4)	162
C12—H12⋯O2^ii^	0.93	2.56	3.308 (4)	138

Symmetry codes: (i) 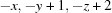; (ii) 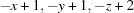.

**Fig. 1.**
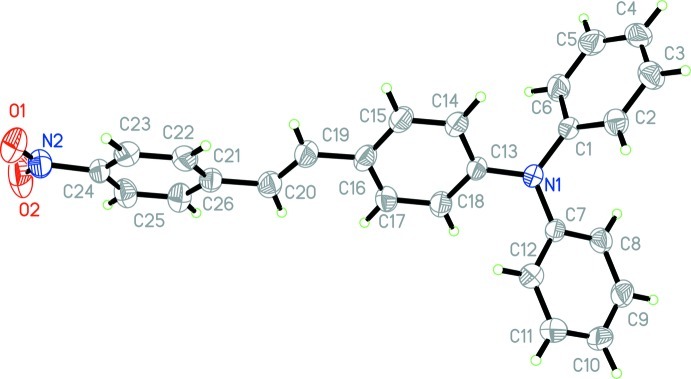
The molecular structure of (I) showing the atomic numbering and 30% probability displacement ellipsoids.

## supplementary crystallographic information

### Comment

Push-pull chromophores are characterized by the conjugated linkage of an electron-donating group (D) and an electron-accepting group (A). Such molecules are potentially useful for nonlinear optical applications and many D-π-A type chromophores have been reported (Marder *et al.*, 1991; Reinhardt *et al.*, 1998). As a part of an ongoing study of such type chromophores, here we report the crystal structure of the title compound, (I).

In (I) (Fig. 1), all bond lengths and angles are normal and correspond to those observed in the related 4-(4-methoxystyryl)-N,N-diphenylaniline (Yang *et al.*, 2003). The C = C double bond ( = 1.276 (4) Å) in the molecule adopts a trans-configuration. The dihedral angle between the benzene ring of the triarylamine group and another benzene ring linked by double bond is 31.34 (13) °. In the crystal, weak intermolecular C—H···O interactions (Table 1) link the molecules into ribbons in [100].

### Experimental

2.73 g (10 mmol) of diethyl(4-nitrophenyl)methylphosphonate were put into a dry mortar, then 2.24 g (20 mmol) t-BuOK were placed into powder, a modicum of 18-crown-6 and 2.72 g (10 mmol) 4-(diphenylamino)benzaldehyde were adde and vigorously grinded. The mixture was monitored by TLC. After completion of the reaction the mixture was dissolved in 150 ml CH_2_Cl_2_ and washed with Di-water, the organic layer separated and dried over MgSO_4_,filterd and solvent removed *in vacuo*. The product was recrystallized from anhydrous ethanol, to give 2.12 g red acicular crystal. Yield, 54.1%. 1H NMR: (400 Hz, CDCl_3_), d(p.p.m.): 8.20(d, 2H, J = 8.4 Hz) 7.60(d, 2H, J=8.8 Hz) 7.40(d, 2H, J=8.8 Hz) 7.30–7.26(m, 4H) 7.19(s, 1H) 7.13(d, 4H, J=8.0) 7.09–7.03(m, 4H) 6.99(s, 1H) 13 C NMR (125 MHz, CDCl_3_) d (p.p.m.) 148.7, 147.8, 147.4, 146.5, 145.0, 144.5, 133.5, 133.0, 130.0 129.7, 129.5, 129.4, 128.2, 126.7, 126.5, 125.1, 124.8, 124.3, 123.8 123.6, 122.8, 122.3. IR (KBr, cm^-1^): 3029 1585 1514 1485 1335 1283 1175 1111 970 841 758 694. MS: m/*z* (%) = 504.20 (100)

### Refinement

All hydrogen atoms were placed in geometrically idealized positions (C—H = 0.93 Å), and constrained to ride on their parent atoms, with *U*_iso_(H) = 1.2 *U*_eq_(C).

### Crystal data

**Table:**

C_26_H_20_N_2_O_2_	*F*(000) = 824
*M**_r_* = 392.44	*D*_x_ = 1.270 Mg m^−^^3^
Monoclinic, *P*2_1_/*c*	Mo *K*α radiation, λ = 0.71073 Å
Hall symbol: -P 2ybc	Cell parameters from 1419 reflections
*a* = 8.4884 (3) Å	θ = 2.4–21.1°
*b* = 8.9834 (3) Å	µ = 0.08 mm^−^^1^
*c* = 27.0880 (8) Å	*T* = 298 K
β = 96.500 (2)°	Red, block
*V* = 2052.31 (12) Å^3^	0.30 × 0.20 × 0.20 mm
*Z* = 4	

### Data collection

**Table:**

Bruker SMART CCD area-detector diffractometer	3606 independent reflections
Radiation source: fine-focus sealed tube	2213 reflections with *I* > 2σ(*I*)
Graphite monochromator	*R*_int_ = 0.026
phi and ω scans	θ_max_ = 25.0°, θ_min_ = 2.4°
Absorption correction: multi-scan (*SADABS*; Sheldrick, 1996)	*h* = −8→10
*T*_min_ = 0.976, *T*_max_ = 0.984	*k* = −10→7
7675 measured reflections	*l* = −32→32

### Refinement

**Table:**

Refinement on *F*^2^	Secondary atom site location: difference Fourier map
Least-squares matrix: full	Hydrogen site location: inferred from neighbouring sites
*R*[*F*^2^ > 2σ(*F*^2^)] = 0.057	H-atom parameters constrained
*wR*(*F*^2^) = 0.185	*w* = 1/[σ^2^(*F*_o_^2^) + (0.0857*P*)^2^ + 0.4264*P*] where *P* = (*F*_o_^2^ + 2*F*_c_^2^)/3
*S* = 1.04	(Δ/σ)_max_ < 0.001
3606 reflections	Δρ_max_ = 0.44 e Å^−^^3^
272 parameters	Δρ_min_ = −0.20 e Å^−^^3^
7 restraints	Extinction correction: *SHELXL97* (Sheldrick, 2008), Fc^*^=kFc[1+0.001xFc^2^λ^3^/sin(2θ)]^-1/4^
Primary atom site location: structure-invariant direct methods	Extinction coefficient: 0.0070 (17)

### Special details

**Table:**

Geometry. All e.s.d.'s (except the e.s.d. in the dihedral angle between two l.s. planes) are estimated using the full covariance matrix. The cell e.s.d.'s are taken into account individually in the estimation of e.s.d.'s in distances, angles and torsion angles; correlations between e.s.d.'s in cell parameters are only used when they are defined by crystal symmetry. An approximate (isotropic) treatment of cell e.s.d.'s is used for estimating e.s.d.'s involving l.s. planes.
Refinement. Refinement of *F*^2^ against ALL reflections. The weighted *R*-factor *wR* and goodness of fit *S* are based on *F*^2^, conventional *R*-factors *R* are based on *F*, with *F* set to zero for negative *F*^2^. The threshold expression of *F*^2^ > σ(*F*^2^) is used only for calculating *R*-factors(gt) *etc*. and is not relevant to the choice of reflections for refinement. *R*-factors based on *F*^2^ are statistically about twice as large as those based on *F*, and *R*- factors based on ALL data will be even larger.

### Fractional atomic coordinates and isotropic or equivalent isotropic displacement parameters (Å^2^)

**Table:**

	*x*	*y*	*z*	*U*_iso_*/*U*_eq_	
O1	0.1022 (4)	0.1217 (4)	1.06736 (14)	0.1466 (14)	
O2	0.2236 (5)	−0.0213 (4)	1.02338 (11)	0.1424 (13)	
N1	0.4781 (2)	1.2271 (2)	0.81947 (8)	0.0608 (6)	
N2	0.1826 (4)	0.1004 (4)	1.03580 (13)	0.0983 (10)	
C1	0.3616 (3)	1.3187 (3)	0.78910 (9)	0.0553 (7)	
C2	0.3483 (3)	1.4677 (3)	0.79922 (10)	0.0654 (7)	
H2	0.4128	1.5106	0.8254	0.078*	
C3	0.2364 (4)	1.5551 (4)	0.76972 (13)	0.0805 (9)	
H3	0.2289	1.6559	0.7770	0.097*	
C4	0.1355 (4)	1.4960 (5)	0.72958 (14)	0.0883 (10)	
H4	0.0631	1.5566	0.7107	0.106*	
C5	0.1463 (4)	1.3478 (4)	0.71906 (13)	0.0938 (11)	
H5	0.0806	1.3053	0.6930	0.113*	
C6	0.2598 (4)	1.2596 (3)	0.74862 (11)	0.0808 (9)	
H6	0.2677	1.1590	0.7411	0.097*	
C7	0.6385 (3)	1.2784 (3)	0.83323 (9)	0.0520 (6)	
C8	0.7061 (3)	1.3620 (3)	0.80040 (11)	0.0685 (8)	
H8	0.6519	1.3862	0.7697	0.082*	
C9	0.8628 (4)	1.4119 (3)	0.81420 (15)	0.0855 (10)	
H9	0.9097	1.4701	0.7915	0.103*	
C10	0.9527 (4)	1.3801 (3)	0.85959 (15)	0.0848 (10)	
H10	1.0551	1.4169	0.8667	0.102*	
C11	0.8878 (4)	1.2964 (3)	0.89204 (12)	0.0769 (8)	
H11	0.9441	1.2712	0.9224	0.092*	
C12	0.7319 (3)	1.2467 (3)	0.87945 (10)	0.0638 (7)	
H12	0.6861	1.1894	0.9026	0.077*	
C13	0.4293 (3)	1.0942 (3)	0.83959 (9)	0.0550 (7)	
C14	0.2812 (3)	1.0854 (3)	0.85408 (10)	0.0634 (7)	
H14	0.2101	1.1638	0.8479	0.076*	
C15	0.2386 (4)	0.9612 (3)	0.87764 (11)	0.0701 (8)	
H15	0.1375	0.9567	0.8877	0.084*	
C16	0.3393 (3)	0.8402 (3)	0.88742 (10)	0.0664 (7)	
C17	0.4834 (3)	0.8447 (3)	0.87023 (11)	0.0679 (7)	
H17	0.5512	0.7634	0.8746	0.081*	
C18	0.5286 (3)	0.9702 (3)	0.84636 (11)	0.0667 (7)	
H18	0.6272	0.9725	0.8345	0.080*	
C19	0.2936 (4)	0.7155 (3)	0.91720 (11)	0.0761 (8)	
H19	0.1950	0.7239	0.9288	0.091*	
C20	0.3702 (4)	0.5964 (3)	0.92954 (11)	0.0729 (8)	
H20	0.4687	0.5873	0.9180	0.088*	
C21	0.3225 (3)	0.4725 (3)	0.95959 (10)	0.0640 (7)	
C22	0.2273 (3)	0.4916 (3)	0.99516 (12)	0.0730 (8)	
H22	0.1908	0.5861	1.0021	0.088*	
C23	0.1839 (3)	0.3702 (4)	1.02138 (12)	0.0793 (9)	
H23	0.1189	0.3830	1.0465	0.095*	
C24	0.2349 (3)	0.2320 (3)	1.01095 (11)	0.0691 (8)	
C25	0.3328 (4)	0.2112 (3)	0.97748 (11)	0.0768 (9)	
H25	0.3709	0.1169	0.9711	0.092*	
C26	0.3760 (4)	0.3325 (3)	0.95259 (11)	0.0752 (8)	
H26	0.4469	0.3190	0.9292	0.090*	

### Atomic displacement parameters (Å^2^)

**Table:**

	*U*^11^	*U*^22^	*U*^33^	*U*^12^	*U*^13^	*U*^23^
O1	0.102 (2)	0.178 (3)	0.165 (3)	−0.0168 (19)	0.041 (2)	0.086 (2)
O2	0.225 (4)	0.0826 (19)	0.114 (2)	−0.039 (2)	−0.005 (2)	0.0338 (17)
N1	0.0531 (13)	0.0507 (13)	0.0774 (15)	−0.0088 (10)	0.0022 (11)	0.0142 (11)
N2	0.093 (2)	0.103 (3)	0.093 (2)	−0.029 (2)	−0.0151 (18)	0.038 (2)
C1	0.0514 (15)	0.0541 (16)	0.0602 (15)	−0.0067 (12)	0.0048 (12)	0.0089 (12)
C2	0.0701 (18)	0.0632 (19)	0.0639 (17)	0.0010 (14)	0.0119 (14)	0.0016 (14)
C3	0.077 (2)	0.076 (2)	0.093 (2)	0.0177 (17)	0.0258 (19)	0.0167 (18)
C4	0.0605 (19)	0.107 (3)	0.098 (3)	0.0091 (18)	0.0143 (18)	0.046 (2)
C5	0.079 (2)	0.110 (3)	0.086 (2)	−0.025 (2)	−0.0210 (18)	0.025 (2)
C6	0.089 (2)	0.0646 (19)	0.084 (2)	−0.0166 (16)	−0.0122 (18)	0.0074 (16)
C7	0.0505 (14)	0.0440 (14)	0.0622 (15)	−0.0018 (11)	0.0101 (12)	0.0037 (12)
C8	0.0616 (17)	0.0667 (18)	0.0794 (19)	0.0054 (14)	0.0177 (15)	0.0196 (15)
C9	0.0581 (18)	0.072 (2)	0.131 (3)	−0.0009 (15)	0.031 (2)	0.0299 (19)
C10	0.0476 (17)	0.069 (2)	0.136 (3)	−0.0007 (15)	0.0042 (19)	0.010 (2)
C11	0.0645 (19)	0.076 (2)	0.086 (2)	−0.0012 (16)	−0.0068 (16)	0.0027 (17)
C12	0.0594 (16)	0.0646 (17)	0.0673 (17)	−0.0032 (13)	0.0075 (13)	0.0034 (13)
C13	0.0568 (16)	0.0473 (15)	0.0599 (15)	−0.0101 (12)	0.0029 (12)	0.0041 (11)
C14	0.0604 (17)	0.0576 (17)	0.0724 (18)	−0.0102 (13)	0.0086 (14)	0.0010 (13)
C15	0.0655 (18)	0.0692 (19)	0.0760 (19)	−0.0166 (15)	0.0093 (15)	0.0024 (15)
C16	0.0727 (16)	0.0654 (16)	0.0598 (16)	−0.0262 (15)	0.0022 (13)	0.0002 (12)
C17	0.0739 (17)	0.0495 (16)	0.0790 (19)	−0.0023 (13)	0.0024 (14)	0.0040 (13)
C18	0.0679 (18)	0.0557 (17)	0.0778 (18)	−0.0030 (14)	0.0136 (14)	0.0061 (14)
C19	0.078 (2)	0.0675 (16)	0.080 (2)	−0.0169 (13)	−0.0011 (15)	0.0085 (14)
C20	0.081 (2)	0.0664 (16)	0.0682 (18)	−0.0164 (13)	−0.0052 (15)	0.0025 (13)
C21	0.0659 (17)	0.0672 (17)	0.0566 (16)	−0.0140 (14)	−0.0033 (14)	0.0054 (12)
C22	0.0665 (18)	0.0613 (18)	0.089 (2)	0.0021 (14)	−0.0002 (16)	0.0082 (16)
C23	0.0585 (18)	0.094 (3)	0.085 (2)	−0.0007 (17)	0.0104 (16)	0.0212 (19)
C24	0.0645 (18)	0.072 (2)	0.0670 (18)	−0.0163 (16)	−0.0075 (15)	0.0249 (15)
C25	0.103 (2)	0.0576 (18)	0.0674 (18)	−0.0071 (16)	−0.0012 (18)	0.0044 (15)
C26	0.095 (2)	0.068 (2)	0.0633 (17)	−0.0131 (17)	0.0124 (16)	−0.0006 (15)

### Geometric parameters (Å, º)

**Table:**

O1—N2	1.168 (4)	C12—H12	0.9300
O2—N2	1.207 (4)	C13—C14	1.361 (4)
N1—C13	1.395 (3)	C13—C18	1.397 (4)
N1—C7	1.445 (3)	C14—C15	1.355 (4)
N1—C1	1.465 (3)	C14—H14	0.9300
N2—C24	1.454 (4)	C15—C16	1.390 (4)
C1—C2	1.373 (4)	C15—H15	0.9300
C1—C6	1.420 (4)	C16—C17	1.358 (4)
C2—C3	1.409 (4)	C16—C19	1.458 (4)
C2—H2	0.9300	C17—C18	1.375 (4)
C3—C4	1.410 (5)	C17—H17	0.9300
C3—H3	0.9300	C18—H18	0.9300
C4—C5	1.367 (5)	C19—C20	1.276 (4)
C4—H4	0.9300	C19—H19	0.9300
C5—C6	1.422 (4)	C20—C21	1.463 (4)
C5—H5	0.9300	C20—H20	0.9300
C6—H6	0.9300	C21—C22	1.337 (4)
C7—C8	1.342 (3)	C21—C26	1.358 (4)
C7—C12	1.433 (4)	C22—C23	1.374 (4)
C8—C9	1.413 (4)	C22—H22	0.9300
C8—H8	0.9300	C23—C24	1.356 (4)
C9—C10	1.401 (5)	C23—H23	0.9300
C9—H9	0.9300	C24—C25	1.310 (4)
C10—C11	1.323 (4)	C25—C26	1.354 (4)
C10—H10	0.9300	C25—H25	0.9300
C11—C12	1.402 (4)	C26—H26	0.9300
C11—H11	0.9300		
			
C13—N1—C7	119.0 (2)	C14—C13—N1	119.2 (2)
C13—N1—C1	119.2 (2)	C14—C13—C18	118.6 (2)
C7—N1—C1	121.44 (19)	N1—C13—C18	122.2 (2)
O1—N2—O2	124.3 (4)	C15—C14—C13	119.2 (3)
O1—N2—C24	116.1 (4)	C15—C14—H14	120.4
O2—N2—C24	119.6 (4)	C13—C14—H14	120.4
C2—C1—C6	117.5 (3)	C14—C15—C16	122.9 (3)
C2—C1—N1	120.0 (2)	C14—C15—H15	118.6
C6—C1—N1	122.5 (2)	C16—C15—H15	118.6
C1—C2—C3	119.7 (3)	C17—C16—C15	118.0 (3)
C1—C2—H2	120.1	C17—C16—C19	121.3 (3)
C3—C2—H2	120.1	C15—C16—C19	120.6 (3)
C2—C3—C4	122.6 (3)	C16—C17—C18	119.7 (3)
C2—C3—H3	118.7	C16—C17—H17	120.1
C4—C3—H3	118.7	C18—C17—H17	120.1
C5—C4—C3	118.6 (3)	C17—C18—C13	121.3 (3)
C5—C4—H4	120.7	C17—C18—H18	119.4
C3—C4—H4	120.7	C13—C18—H18	119.4
C4—C5—C6	118.8 (3)	C20—C19—C16	129.0 (3)
C4—C5—H5	120.6	C20—C19—H19	115.5
C6—C5—H5	120.6	C16—C19—H19	115.5
C1—C6—C5	122.7 (3)	C19—C20—C21	128.2 (3)
C1—C6—H6	118.6	C19—C20—H20	115.9
C5—C6—H6	118.6	C21—C20—H20	115.9
C8—C7—C12	117.0 (2)	C22—C21—C26	117.3 (3)
C8—C7—N1	118.0 (2)	C22—C21—C20	122.1 (3)
C12—C7—N1	125.0 (2)	C26—C21—C20	120.6 (3)
C7—C8—C9	117.6 (3)	C21—C22—C23	119.4 (3)
C7—C8—H8	121.2	C21—C22—H22	120.3
C9—C8—H8	121.2	C23—C22—H22	120.3
C10—C9—C8	124.7 (3)	C24—C23—C22	120.6 (3)
C10—C9—H9	117.6	C24—C23—H23	119.7
C8—C9—H9	117.6	C22—C23—H23	119.7
C11—C10—C9	118.2 (3)	C25—C24—C23	121.2 (3)
C11—C10—H10	120.9	C25—C24—N2	117.1 (3)
C9—C10—H10	120.9	C23—C24—N2	121.8 (3)
C10—C11—C12	118.2 (3)	C24—C25—C26	117.3 (3)
C10—C11—H11	120.9	C24—C25—H25	121.4
C12—C11—H11	120.9	C26—C25—H25	121.4
C11—C12—C7	124.3 (3)	C25—C26—C21	124.2 (3)
C11—C12—H12	117.9	C25—C26—H26	117.9
C7—C12—H12	117.9	C21—C26—H26	117.9
			
C13—N1—C1—C2	129.1 (3)	C18—C13—C14—C15	4.5 (4)
C7—N1—C1—C2	−44.5 (3)	C13—C14—C15—C16	−0.7 (4)
C13—N1—C1—C6	−51.1 (3)	C14—C15—C16—C17	−3.4 (4)
C7—N1—C1—C6	135.3 (3)	C14—C15—C16—C19	173.8 (3)
C6—C1—C2—C3	0.0 (4)	C15—C16—C17—C18	3.5 (4)
N1—C1—C2—C3	179.7 (2)	C19—C16—C17—C18	−173.8 (3)
C1—C2—C3—C4	0.1 (4)	C16—C17—C18—C13	0.4 (4)
C2—C3—C4—C5	0.3 (5)	C14—C13—C18—C17	−4.5 (4)
C3—C4—C5—C6	−0.6 (5)	N1—C13—C18—C17	174.2 (2)
C2—C1—C6—C5	−0.4 (4)	C17—C16—C19—C20	−3.1 (5)
N1—C1—C6—C5	179.9 (3)	C15—C16—C19—C20	179.7 (3)
C4—C5—C6—C1	0.7 (5)	C16—C19—C20—C21	179.8 (3)
C13—N1—C7—C8	151.6 (2)	C19—C20—C21—C22	−29.6 (5)
C1—N1—C7—C8	−34.8 (3)	C19—C20—C21—C26	151.3 (3)
C13—N1—C7—C12	−28.3 (4)	C26—C21—C22—C23	−2.3 (4)
C1—N1—C7—C12	145.3 (2)	C20—C21—C22—C23	178.6 (3)
C12—C7—C8—C9	−0.2 (4)	C21—C22—C23—C24	−0.9 (4)
N1—C7—C8—C9	179.9 (2)	C22—C23—C24—C25	3.4 (5)
C7—C8—C9—C10	0.2 (5)	C22—C23—C24—N2	−175.9 (3)
C8—C9—C10—C11	0.6 (5)	O1—N2—C24—C25	176.1 (3)
C9—C10—C11—C12	−1.2 (5)	O2—N2—C24—C25	−3.6 (5)
C10—C11—C12—C7	1.1 (4)	O1—N2—C24—C23	−4.6 (5)
C8—C7—C12—C11	−0.4 (4)	O2—N2—C24—C23	175.7 (3)
N1—C7—C12—C11	179.5 (2)	C23—C24—C25—C26	−2.3 (5)
C7—N1—C13—C14	139.6 (3)	N2—C24—C25—C26	177.0 (3)
C1—N1—C13—C14	−34.2 (3)	C24—C25—C26—C21	−1.2 (5)
C7—N1—C13—C18	−39.1 (4)	C22—C21—C26—C25	3.5 (4)
C1—N1—C13—C18	147.1 (3)	C20—C21—C26—C25	−177.4 (3)
N1—C13—C14—C15	−174.2 (2)		

### Hydrogen-bond geometry (Å, º)

**Table:**

*D*—H···*A*	*D*—H	H···*A*	*D*···*A*	*D*—H···*A*
C15—H15···O1^i^	0.93	2.58	3.481 (4)	162
C12—H12···O2^ii^	0.93	2.56	3.308 (4)	138

Symmetry codes: (i) −*x*, −*y*+1, −*z*+2; (ii) −*x*+1, −*y*+1, −*z*+2.
